# Supplementary material for: Stomoxys Species Richness and Apparent Densities at Different Land-Use Setups in North-Eastern KwaZulu-Natal Province, South Africa
Source: Insects. 2025 Oct 15;16(10):1049. doi: 10.3390/insects16101049 (PMC12564159; doi:10.3390/insects16101049)
Supplement: Supplementary file 1 [file insects-16-01049-s001.zip › insects-3837616-supplementary.pdf]

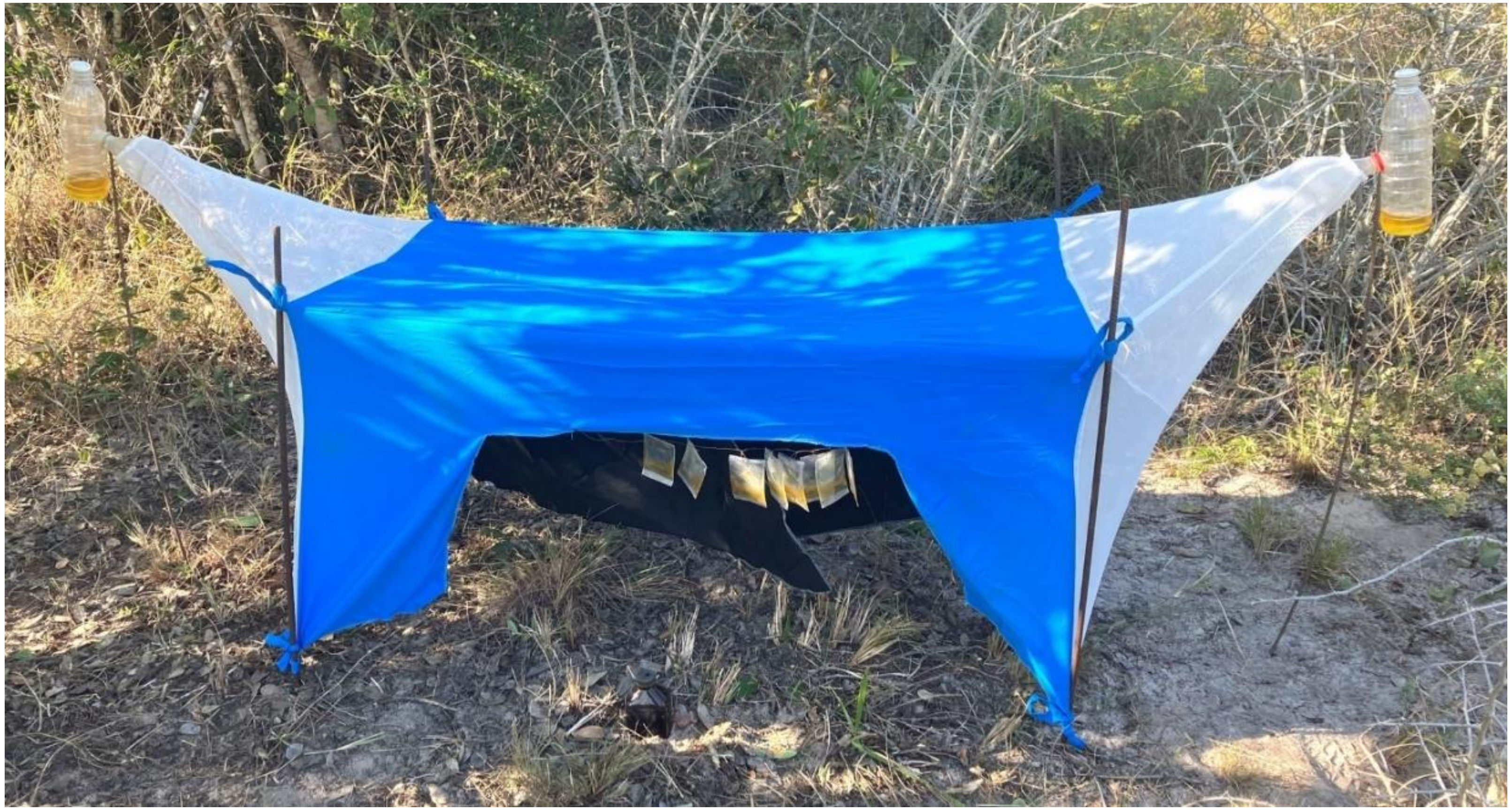

Supplementary file 1: Fig. S1

H-traps used to sample for *Stomoxys* flies across the three different ecological settings in northeastern KwaZulu-Natal

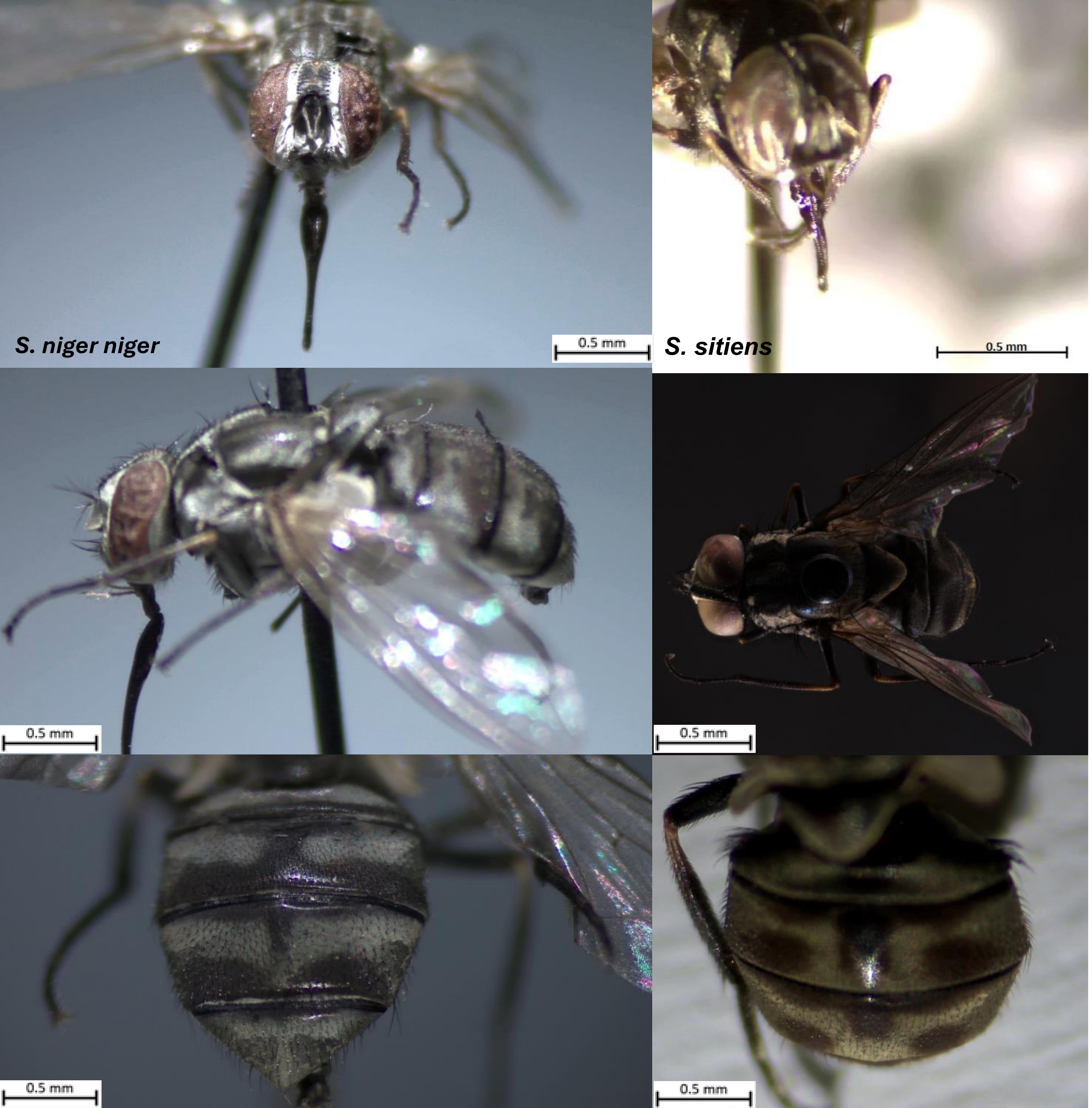

Supplementary file 2: Fig. S2

Six *Stomoxys* species collected from the three ecological settings in north-eastern KwaZulu-Natal Province, South Africa

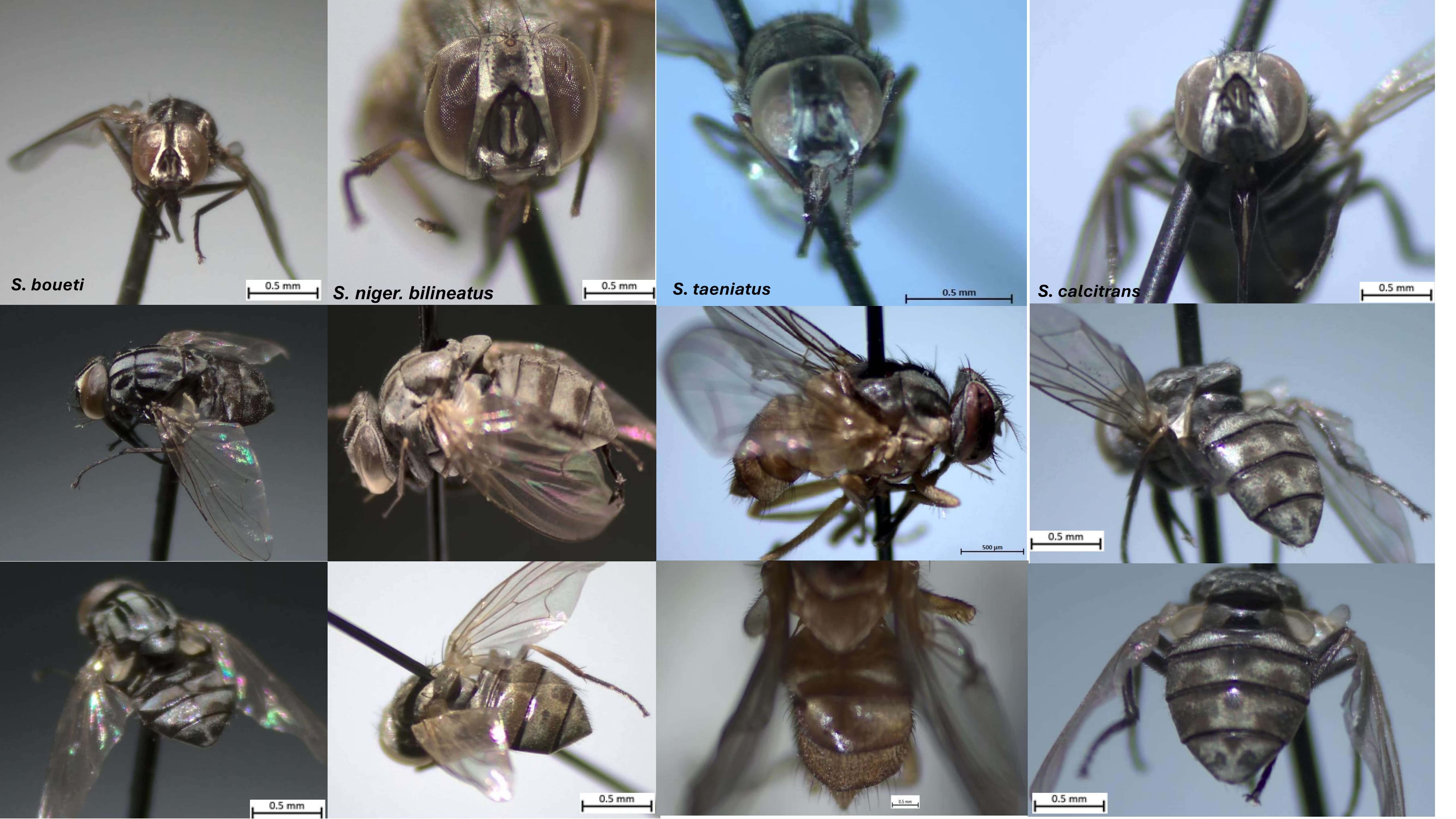
S. boueti
0.5 mm
S. niger. bilineatus
0.5 mm
S. taeniatus
0.5 mm
S. calcitrans
0.5 mm
500 µm
0.5 mm
0.5 mm
0.5 mm
0.5 mm
0.5 mm
